# Dynamic biomarker trajectories in the first 72 h after infarct-related cardiac arrest: a novel approach to early risk stratification

**DOI:** 10.1016/j.resplu.2025.101126

**Published:** 2025-10-13

**Authors:** Julian Mohsennia, Sophia Neschen, Joshua Boettel, Steffen Desch, Youssef Abdelwahed, Tobias Petzold, Andi Rroku, Eva-Maria Dorsch, Georg Girke, Benjamin O’Brien, Ulf Landmesser, Carsten Skurk, Tharusan Thevathasan

**Affiliations:** a Department of Cardiology, Angiology and Intensive Care Medicine, Deutsches Herzzentrum der Charité (DHZC), Campus Benjamin Franklin, Berlin, Germany; b Department of Internal Medicine/Cardiology, Heart Center Leipzig at the University of Leipzig, Leipzig, Germany; c Department of Psychosomatic Medicine, Charité - Universitätsmedizin Berlin, Berlin, Germany; d Department of Cardiac Anesthesiology and Intensive Care Medicine, Deutsches Herzzentrum der Charité, Berlin, Germany; e Berlin Institute of Health, Berlin, Germany; f Deutsches Zentrum für Herz-Kreislauf-Forschung, Partner Site Berlin, Berlin, Germany

**Keywords:** Cardiac arrest, Acute myocardial infarction, Biomarkers, Risk stratification, Multi-organ dysfunction, Post-resuscitation care

## Abstract

**Background:**

Cardiac arrest caused by acute myocardial infarction (AMI) is associated with high mortality. Although risk stratification scores exist, they rely primarily on static variables obtained at admission, which do not capture the dynamic pathophysiology of the post-resuscitation phase. This study aimed to evaluate the prognostic value of serial biomarker trajectories during the first 72 h after AMI-induced cardiac arrest.

**Methods:**

In this single-center cohort study, 181 patients with AMI-induced cardiac arrest between 2018 and 2024 were analyzed. Routinely measured laboratory biomarkers were assessed over the first three days in the intensive care unit (ICU). Multivariable logistic regression models adjusted for key clinical covariates were used to evaluate associations between biomarker trajectories and in-hospital mortality. Secondary analyses included t-distributed stochastic neighbor embedding cluster (machine learning), radar, Sankey and trend plots to visualize biomarker patterns in survivors and non-survivors.

**Results:**

Of the 181 patients, 65.2% survived to hospital discharge. Survivors and non-survivors showed overlapping biomarker profiles on day one, with clearer separation emerging by day three. Non-survivors demonstrated progressive multi-organ dysfunction, including elevated levels of creatinine, potassium, creatine kinase, lactate, neuron-specific enolase, leukocytes and persistent coagulopathy, while survivors showed restoration of physiological homeostasis. Several biomarkers and their dynamic changes over 72 h independently predicted mortality. Cluster, radar, Sankey and trend plot analyses supported the concept of diverging physiological trajectories between survivors and non-survivors over time.

**Conclusions:**

In patients who survive the initial critical phase after cardiac arrest, early prognostication remains limited due to evolving clinical trajectories. Admission biomarkers alone are insufficient for making definitive decisions. The post-resuscitation period represents a critical “second hit” characterized by systemic inflammation and organ dysfunction. Integrating serial biomarker trends into dynamic risk models, such as with machine learning, offers a more individualized and accurate approach to post-cardiac arrest prognostication and care.

## Graphical abstract

***Central Illustration.*** Trajectory of biomarker levels following infarct-related cardiac arrest. (A) Radar plots illustrate the relationship between biomarker levels and in-hospital outcomes from day one to day three following infarct-related cardiac arrest. The dashed black line represents the overall cohort mean (set to 0), with each concentric grey ring denoting 0.1 standard deviation increments. Biomarker values for survivors are shown as a green line, while those for non-survivors are shown in red. Deviations above the cohort mean extend beyond the dashed line, while below-average values fall within it, allowing visual comparison of multivariate biomarker profiles over time. (B) The t-distributed stochastic neighbor embedding (t-SNE) algorithm is a dimensionality reduction technique that transforms complex, high-dimensional biomarker data into a two-dimensional space for visual interpretation. By estimating the pairwise similarity of patients based on biomarker patterns, t-SNE projects them so that clinically similar profiles are positioned closer together, while dissimilar profiles are spaced further apart. In this plot, each dot represents an individual patient, colored by hospital outcome (green: survival, red: death). Clustering was performed using biomarker values from day 1 and day 3, revealing distinct patterns and outcome-related separations across the study population.

**Figure:**
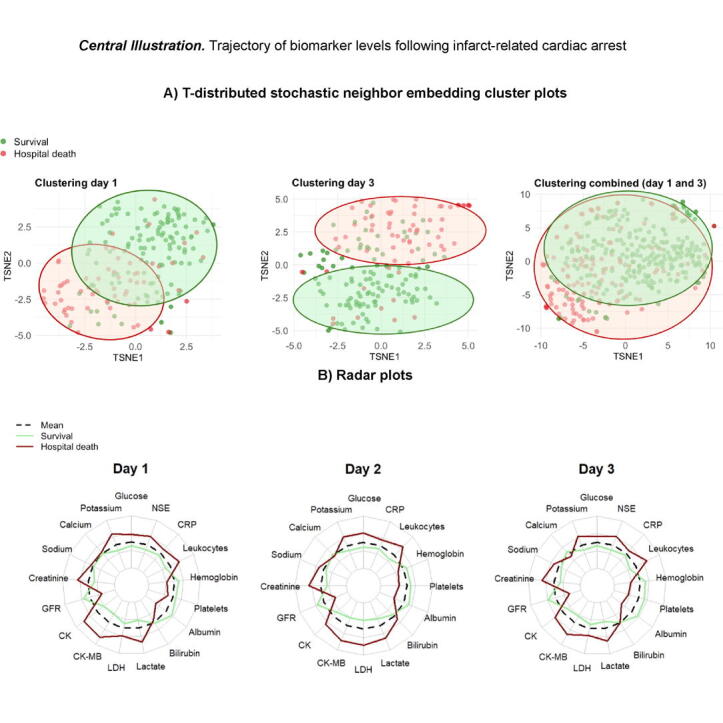

## Introduction

Cardiac arrest remains one of the leading causes of death worldwide, and despite advances in resuscitation and critical care, survival rates remain as low as 18 %.^1^, ^2^ Among the etiologies of cardiac arrest, acute myocardial infarction (AMI) stands out as a predominant etiology.^3^ Patients who experience cardiac arrest in the context of AMI are particularly considered high-risk due to the combined burden of myocardial injury, systemic hypoperfusion and potential for recurrent ischemia.

To date, a number of clinical risk stratification scores, such as the “OHCA”, “CAHP”, “TTM”, “MIRACLE_2_” and “TOMAHAWK” scores, have been developed to predict outcomes in cardiac arrest survivors.^4^ These tools, however, are predominantly constructed using static variables obtained at the time of arrest or upon hospital admission, such as age, initial rhythm, no-flow and low-flow times as well as basic laboratory parameters. While useful for early orientation, these scores may fail to reflect the complex dynamic nature of post-resuscitation injury. Crucially, neurological prognostication is generally deferred until 72 h after cardiac arrest,^5^ by which time many admission parameters may no longer accurately reflect the patient’s clinical status. This temporal mismatch raises concern that early prognostic decisions based solely on initial data may misclassify patients with reversible pathology (such as AMI) or evolving recovery.

The post-resuscitation period, often referred to as the “second hit,” is increasingly recognized as a critical window during which systemic inflammation, ischemia–reperfusion injury and multi-organ dysfunction may unfold − processes that directly influence survival.^6^ Therefore, focusing exclusively on admission-based predictors risks may oversimplify the trajectory of critically ill patients. Dynamic assessment strategies that capture changes in clinical and biochemical parameters over time may provide more robust and nuanced prognostic information.^7^

Given these considerations, the temporal evolution of biomarkers which reflect the underlying pathophysiology of the post-cardiac arrest syndrome may prove advantageous. Routinely measured laboratory values, when assessed longitudinally, may provide critical insights into patient trajectories and inform the timing and intensity of interventions. The objective of this study was to analyze the clustering and trajectory patterns of routine biomarkers over the first 72 h following AMI-induced cardiac arrest and to identify those parameters that independently predict in-hospital mortality. By integrating dynamic biomarker data with clinical outcomes by using modern machine learn approaches, this study aims to advance risk stratification and support a more individualized, physiology-driven approach to post-resuscitation care.

## Material and methods

### Study population

The study was conducted at a single tertiary cardiac care center at the Deutsches Herzzentrum der Charité in Berlin, Germany. Ethical approval was obtained from the institutional review board (protocol number: EA4/220/21). The study adheres to the Strengthening the Reporting of Observational Studies in Epidemiology (STROBE) guidelines and was conducted in accordance with the principles outlined in the Declaration of Helsinki by the World Medical Association. Analyses were performed within a consecutively enrolled cohort of adult patients who experienced AMI-induced cardiac arrest, including both out-of-hospital cardiac arrest (OHCA) and in-hospital cardiac arrest (IHCA), between 2018 and 2024. AMI was defined as the presence of a significant coronary artery stenosis identified during coronary angiography, consistent with type 1 AMI, which was immediately treated by percutaneous coronary intervention (PCI).^8^ A subgroup of patients in the cohort received extracorporeal cardiopulmonary resuscitation (ECPR) for refractory cardiac arrest. ECPR was delivered using veno-arterial extracorporeal membrane oxygenation (VA-ECMO), either as a standalone modality or in combination with a micro-axial flow pump (mAFP), in what is referred to as the “ECMELLA” approach.^9^, ^10^ All included patients underwent immediate PCI during the index event in accordance with current guideline recommendations of the European Society of Cardiology. No patients were referred for CABG during the acute phase.^11^

### Data sources and data collection

Four institutional databases were used to extract comprehensive hospital data for this study. The Intensive Care Electronic Patient Information System (COPRA System GmbH) was used to obtain detailed data from the intensive care unit (ICU), while Centricity™ RIS-i (GE Healthcare) provided information related to interventional procedures. Patient demographics and hospital-related variables were retrieved from the SAP® ishmed® hospital information system and emergency medical service protocols were reviewed to extract prehospital cardiac arrest parameters, as previously described in the literature.^12^ All data entries were manually validated by three independent investigators through a detailed review of discharge summaries and clinical notes to ensure accuracy and completeness.

The variables collected included a comprehensive range of patient characteristics and clinical parameters: Demographic data included age, sex and body mass index (BMI), while the medical history was assessed using the Charlson Comorbidity Index (CCI), a validated tool that quantifies comorbidity burden.^13^ Specific pre-existing conditions recorded included coronary artery disease (CAD), prior PCI, coronary artery bypass grafting (CABG) or myocardial infarction, previous cardiac arrest, dyslipidemia, diabetes mellitus, hypertension, smoking status and obesity. Detailed information on cardiac arrest characteristics was obtained, including initial arrest rhythm, location of arrest (OHCA vs. IHCA), whether the arrest was witnessed, performance of bystander cardiopulmonary resuscitation (CPR), administration of adrenaline or amiodarone in the prehospital setting as well as “no-flow” and “low-flow” times. Additional resuscitation-related data included whether ECPR was performed and the time from arrest to ECPR initiation. In-hospital treatment data included the location of the coronary culprit lesion, left ventricular ejection fraction (LVEF) after hospital admission and close to discharge or death, as well as the use and duration of VA-ECMO and/or a mAFP. Patient outcomes were meticulously documented, including survival status, neurological outcomes as assessed by the Cerebral Performance Category (CPC) scale and length of stay (LOS) in both the ICU and hospital. A wide spectrum of procedural complications was evaluated, such as major bleeding, the number of administered erythrocyte and thrombocyte units, ischemic events (abdominal, urogenital or limb), abdominal compartment syndrome, pulmonary embolism, hypoxic brain injury, cardiac tamponade, hemolysis, ischemic or hemorrhagic stroke and the requirement for renal replacement therapy (RRT). Major bleeding was defined according to the Bleeding Academic Research Consortium (BARC) criteria, encompassing BARC types 3, 4 and 5.^14^ This includes overt bleeding accompanied by a hemoglobin drop of ≥3 g/dL, intracranial hemorrhage, CABG-related bleeding or fatal bleeding. All complications were identified as part of routine clinical management, utilizing an integrated approach that included clinical assessment, laboratory diagnostics and imaging modalities.

### Early laboratory biomarkers within 72 h after cardiac arrest

Routinely measured laboratory biomarkers were extracted retrospectively from protocolized daily ICU blood draws over three consecutive days following cardiac arrest. All routinely assessed biomarkers were included in the analysis to evaluate their association with in-hospital mortality. The panel of laboratory parameters assessed in this study encompassed a wide range of routinely measured biomarkers reflecting various organ systems. These included blood count and coagulation markers (hemoglobin, platelet count and lactate dehydrogenase [LDH]), inflammatory markers (C-reactive protein [CRP] and leukocyte count), cardiac injury and perfusion markers (creatine kinase [CK], CK-MB and lactate), renal function indicators (serum creatinine and estimated glomerular filtration rate [GFR]), metabolic parameters (glucose, albumin and bilirubin), electrolytes (sodium, potassium and calcium) and neuronal injury markers (neuron-specific enolase [NSE]). Biomarkers were assessed using blood samples collected through pre-existing arterial cannula access.

### Statistical analysis

Data analyses were performed using R Core Team 2020 (Vienna, Austria). Continuous variables were compared using the T-test and results are reported as medians with interquartile ranges (IQR). Categorical variables are presented as frequencies and percentages.

Missing laboratory values were addressed using predictive mean matching, a robust imputation technique that preserves the original data distribution by replacing missing entries with observed values from similar cases based on predicted means. The primary outcome was defined as in-hospital mortality following the cardiac arrest event. Statistical results are expressed as adjusted odds ratios (ORs) with 95 % confidence intervals (CIs) and corresponding p-values. A two-tailed p-value <0.05 was considered statistically significant.

The primary analysis aimed to identify independent predictors of in-hospital mortality among laboratory biomarkers collected during the first 72 h following the cardiac arrest event as well as their absolute and relative changes. Biomarker change refers to the assessment of biomarker levels between hospital days one and three, and serves as an indicator of both biomarker production and metabolic turnover, thereby reflecting the extent of recovery. Absolute change was defined as the numerical difference in biomarker concentrations between the time of measurement at 72 h and the initial value obtained immediately after the cardiac arrest event. In contrast, relative change was defined as the percentage change in biomarker levels at 72 h, calculated in relation to the baseline measurement obtained immediately post-arrest. All aforementioned routinely measured biomarkers were included in multivariable regression models. These models were adjusted for predefined confounders, including age, sex, “low-flow” time and initial arrest rhythm (shockable vs. non-shockable). The selection of these confounders was based on expert consensus from a panel of cardiology consultants with fellowship training in critical care medicine, emergency medicine and/or interventional cardiology. Additionally, a focused review of the medical literature was conducted to incorporate previously validated predictors of post-cardiac arrest survival. In addition, the Youden Index was calculated for each laboratory biomarker, including its absolute and relative changes over 72 h, to determine the optimal cut-off value and to assess the diagnostic efficacy of the biomarker. The Youden Index, based on sensitivity and specificity parameters, quantifies the biomarkeŕs ability to maximize true positives while minimizing false positives and false negatives, thereby providing a single metric to evaluate the discriminatory performance of each laboratory value in predicting in-hospital mortality.

As secondary analyses, cluster plot, radar plot, Sankey plot and time-dependent trend analyses were generated to explore the differential effects of biomarker levels on day one and day three after cardiac arrest between survivors and non-survivors. Cluster plot analyses provide a visual representation that groups biomarker levels of survivors and non-survivors based on similarities across multiple biomarkers. These plots, which use machine learning techniques with t-distributed stochastic neighbor embedding, reveal distinct patient clusters that may reflect divergent physiological responses to cardiac arrest on days one and day three by reducing complex, high-dimensional data to two dimensions for visualization.

Radar plots are circular graphs in which each axis represents a single biomarker, allowing the comparison of multivariable biomarker profiles between surviving and deceased patients at different time points. This format allows for an intuitive visual assessment of complex biomarker interactions. Sankey plots are flow diagrams that show the distribution and transitions of biomarker levels among patients who survived or died. The width of each connecting flow is proportional to the number of patients transitioning between survival and death status, thereby illustrating the magnitude and direction of changes in biomarker profiles across clinical outcomes.

### Subgroup analyses

The primary analyses were repeated in subgroups of patients who did and did not undergo ECPR in order to eliminate the potential confounding effects of refractory cardiac arrest and device-related influences on the observed outcomes.

## Results

### Patient and treatment characteristics

**Fig. 1:**
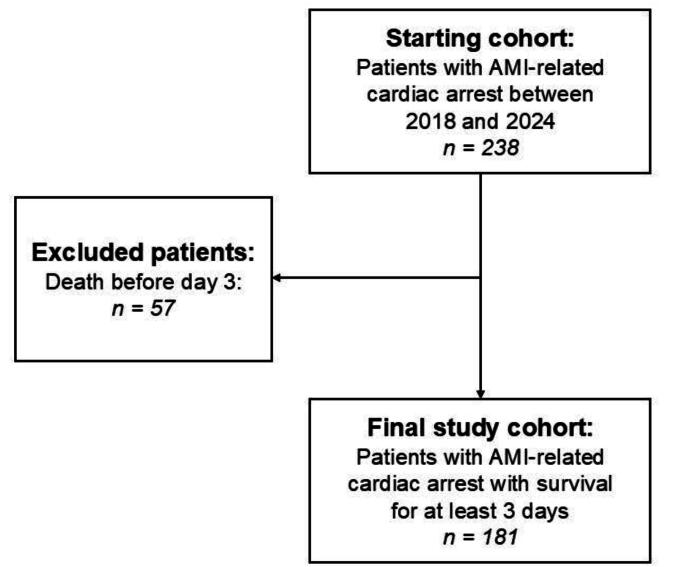
Study flow diagram. Between 2018 and 2024, a total of 332 patients with cardiac arrest were assessed for eligibility. Following the exclusion of 151 individuals, a final cohort of 181 patients with acute myocardial infarction-induced cardiac arrest who survived beyond the initial 72 h was included in the analysis. AMI, acute myocardial infarction.

Between 2018 and 2024, a total of 238 adult patients who experienced AMI-induced cardiac arrest due to AMI were screened for eligibility. Of these, 181 patients met the inclusion criteria and were included in the final analysis. Patients who died before day three were excluded (Fig. 1).

Of the 181 patients included in the study, 118 individuals (65.2 %) survived to hospital discharge. The median age of the study population was 61 years [interquartile range (IQR) 54–75] and 145 patients (80.1 %) were male. The median CCI was 0 [IQR 0–1]. Dyslipidemia (48.6 %), hypertension (60.8 %) and obesity (19.9 %) were relatively common in the cohort prior to the cardiac arrest event. A total of 127 patients (70.2 %) experienced OHCA and 54 patients (29.8 %) experienced IHCA. The median “low-flow” time was 17 min [5–35]. An initial shockable rhythm was present in 149 patients (82.3 %) and, among OHCA cases, 106 (83.5 % of all OHCA cases) were witnessed events, with immediate bystander CPR performed in 108 patients (85 % of all OHCA cases). ECPR was performed in 43 patients (23.8 %), as detailed in Table 1.

**Table 1:** Patient and treatment characteristics.

	**Survival(N = 118)**	**In-hospital death (N = 63)**	**All patients(N = 181)**	**P-value**
**Demographic data**				
**Age (years)**	61.5 [54.3–72.0]	61.0 [54.0–77.0]	61.0 [54.0–75.0]	0.453
**Male Sex**	91 (77.1 %)	54 (85.7 %)	145 (80.1 %)	0.236
**BMI (kg/m^2^)**	26.0 [23.7–27.8]	26.1 [24.2–27.8]	26.0 [24.2–27.8]	0.295

**Past medical history**				
**Charlson Comorbidity Index**	0 [0–1.0]	0 [0–1.0]	0 [0–1.0]	0.916
**CAD degree**				
None	95 (80.5 %)	48 (76.2 %)	143 (79.0 %)	0.336
1-vessel	6 (5.1 %)	1 (1.6 %)	7 (3.9 %)	
2-vessel	7 (5.9 %)	2 (3.2 %)	9 (5.0 %)	
3-vessel	7 (5.9 %)	7 (11.1 %)	14 (7.7 %)	
**Prior PCI**	19 (16.1 %)	11 (17.5 %)	30 (16.6 %)	0.981
**Prior CABG**	4 (3.4 %)	3 (4.8 %)	7 (3.9 %)	0.959
**Prio myocardial infarction**	13 (11.0 %)	7 (11.1 %)	20 (11.0 %)	0.999
**Prior cardiac arrest**	4 (3.4 %)	2 (3.2 %)	6 (3.3 %)	0.999
**Dyslipidemia**	69 (58.5 %)	19 (30.2 %)	88 (48.6 %)	<0.001
**Diabetes mellitus**	13 (11.0 %)	11 (17.5 %)	24 (13.3 %)	0.323
**Hypertension**	70 (59.3 %)	40 (63.5 %)	110 (60.8 %)	0.698
**Smoker**	50 (42.4 %)	19 (30.2 %)	69 (38.1 %)	0.147
**Obesity**	23 (19.5 %)	13 (20.6 %)	36 (19.9 %)	0.969

**Cardiac arrest parameters**				
**Initial arrest Rhythm**				
Ventricular fibrillation	98 (83.1 %)	38 (60.3 %)	136 (75.1 %)	0.003
Ventricular tachycardia	8 (6.8 %)	5 (7.9 %)	13 (7.2 %)	
Asystole	5 (4.2 %)	6 (9.5 %)	11 (6.1 %)	
Pulseless electrical activity	7 (5.9 %)	14 (22.2 %)	21 (11.6 %)	
**Shockable arrest rhythm**	106 (89.8 %)	43 (68.3 %)	149 (82.3 %)	<0.001
**Location of cardiac arrest**				
OHCA	81 (68.6 %)	46 (73.0 %)	127 (70.2 %)	0.659
IHCA	37 (31.4 %)	17 (27.0 %)	54 (29.8 %)	
**For OHCA cases only:** **Witnessed cardiac arrest**	71 (87.7 %)	35 (76.1 %)	106 (83.5 %)	0.611
**For OHCA cases only:** **Bystander CPR performed**	71 (87.7 %)	37 (80.4 %)	108 (85.0 %)	0.924
**Time from arrest to hospital (minutes)**	0 [0–0]	0 [0–55.0]	0 [0–39.0]	0.248
**Time from arrest to CPR** **(“no-flow” time, minutes)**	0 [0–0.75]	0 [0–0]	0 [0–0]	0.551
**Time from arrest to ROSC** **(“low-flow” time, minutes)**	15.0 [3.0–25.0]	30.0 [10.5–55.0]	17.0 [5.0–35.0]	<0.001
**ECPR performed**	15 (12.7 %)	28 (44.4 %)	43 (23.8 %)	<0.001
**For ECPR patients only:** **Time from arrest to ECPR** **(minutes)**	50.0 [31.5–85.0]	73.5 [61.8–90.0]	70.0 [44.0–90.0]	0.496
**Prehospital adrenaline administration**	43 (36.4 %)	35 (55.6 %)	78 (43.1 %)	0.023
**Prehospital amiodaron administration**	31 (26.3 %)	24 (38.1 %)	55 (30.4 %)	0.149

**In-hospital treatment**				
**Current CAD**				
1-vessel	39 (33.1 %)	16 (25.4 %)	55 (30.4 %)	0.021
2-vessel	40 (33.9 %)	13 (20.6 %)	53 (29.3 %)	
3-vessel	39 (33.1 %)	34 (54.0 %)	73 (40.3 %)	
**Culprit lesion**				
LAD	60 (50.8 %)	34 (54.0 %)	94 (51.9 %)	0.066
LM	4 (3.4 %)	5 (7.9 %)	9 (5.0 %)	
RCA	39 (33.1 %)	15 (23.8 %)	54 (29.8 %)	
LCX	15 (12.7 %)	8 (12.7 %)	23 (12.7 %)	
RIM	0 (0 %)	1 (1.6 %)	1 (0.6 %)	
**LVEF at admission (percent)**	40.0 [30.0–50.0]	30.0 [20.0–40.0]	39.0 [30.0–50.0]	<0.001
**LVEF close to discharge or death (percent)**	51.5 [45.0–60.0]	35.0 [30.0–60.0]	50.0 [40.0–60.0]	0.010
**Therapy with VA-ECMO**	19 (16.1 %)	29 (46.0 %)	48 (26.5 %)	<0.001
**Therapy with mAFP**	19 (16.1 %)	34 (54.0 %)	53 (29.3 %)	<0.001
**Treatment duration with VA-ECMO (days)**	0 [0–0]	0 [0–6.0]	0 [0–2.0]	<0.001
**Treatment duration with mAFP (days)**	0 [0–0]	2.0 [0–5.0]	0 [0–2.0]	<0.001

**Procedural complication**				
**BARC category**				
0	94 (79.7 %)	39 (61.9 %)	133 (73.5 %)	0.041
2	5 (4.2 %)	2 (3.2 %)	7 (3.9 %)	
3a	6 (5.1 %)	7 (11.1 %)	13 (7.2 %)	
3b	8 (6.8 %)	4 (6.3 %)	12 (6.6 %)	
3c	0 (0 %)	3 (4.8 %)	3 (1.7 %)	
4	5 (4.2 %)	6 (9.5 %)	11 (6.1 %)	
**Major bleeding (BARC 3**–**5)**	19 (16.1 %)	20 (31.7 %)	39 (21.5 %)	0.018
**Number of red blood cell units transfused**	0 [0–0]	0 [0–6.0]	0 [0–2.0]	0.016
**Number of thrombocyte units transfused**	0 [0–0]	0 [0–0]	0 [0–0]	0.394
**Hemolysis**	36 (30.5 %)	39 (61.9 %)	75 (41.4 %)	<0.001
**Ischemic stroke**	5 (4.2 %)	4 (6.3 %)	9 (5.0 %)	0.792
**Hemorrhagic stroke**	3 (2.5 %)	5 (7.9 %)	8 (4.4 %)	0.193
**Abdominal ischemia**	3 (2.5 %)	8 (12.7 %)	11 (6.1 %)	0.016
**Abdominal compartment syndrome**	0 (0 %)	1 (1.6 %)	1 (0.6 %)	0.752
**Urogenital ischemia**	0 (0 %)	0 (0 %)	0 (0 %)	NA
**Pulmonary embolism**	2 (1.7 %)	0 (0 %)	2 (1.1 %)	0.770
**Thrombosis in arm or leg**	5 (4.2 %)	2 (3.2 %)	7 (3.9 %)	1.000
**Access-site ischemia**	2 (1.7 %)	9 (14.3 %)	11 (6.1 %)	0.002
**Hypoxic brain damage**	38 (32.2 %)	42 (66.7 %)	80 (44.2 %)	<0.001
**Renal replacement therapy**	13 (11.0 %)	32 (50.8 %)	45 (24.9 %)	<0.001
**Sepsis**	10 (8.5 %)	17 (27.0 %)	27 (14.9 %)	0.002
**Pericardial effusion**	2 (1.7 %)	4 (6.3 %)	6 (3.3 %)	0.219
**Severe arrhythmia**	24 (20.3 %)	12 (19.0 %)	36 (19.9 %)	0.991

**Hospital-level data**				
**Hospital length of stay (days)**	13.0 [8.0–23.3]	8.0 [5.0–17.0]	12.0 [7.0–22.0]	0.001
**ICU length of stay (days)**	8.5 [4.0–19.8]	8.0 [5.0–15.5]	8.0 [4.0–18.0]	0.347

BARC, Bleeding Academic Research Consortium; BMI, body mass index; CABG, coronary artery bypass graft; CAD, coronary artery disease; CPR, cardiopulmonary resuscitation; ECPR, extracorporeal cardiopulmonary resuscitation; IHCA, in-hospital cardiac arrest; LAD, left anterior descending coronary artery; LM, left main coronary artery; LVEF, left-ventricular ejection fraction; mAFP, microaxial flow pump; OHCA, out-of-hospital cardiac arrest; PCI, percutaneous coronary intervention; RCA, right coronary artery; LCX, left circumflex coronary artery; RIM, intermediate coronary artery; VA-ECMO, veno-arterial extracorporeal membrane oxygenation

When comparing survivors and non-survivors, survivors were more likely to present with ventricular fibrillation as the initial arrest rhythm (83.1 % vs. 60.3 %, p = 0.003), whereas patients who died were more likely to present with asystole (9.5 % vs. 4.2 %, p = 0.003) or pulseless electrical activity (22.2 % vs. 5.9 %, p = 0.003). Survivors also had shorter “low-flow” durations (15 vs. 30 min, p < 0.001) and were less likely to require ECPR (12.7 % vs. 44.4 %, p < 0.001). Patients who died exhibited more severe lactic acidosis upon admission, as indicated by lower arterial pH (7.12 vs. 7.25, p < 0.001) and higher serum lactate levels (75.0 vs. 42.0 mg/dL, p < 0.001; Table 2). They also had a significantly higher prevalence of three-vessel CAD identified during immediate coronary angiography (54.0 % vs. 33.1 %, p = 0.021), along with a markedly reduced LVEF at both hospital admission (30.0 % vs. 40.0 %, p < 0.001) and close to discharge or death (35.0 % vs. 51.5 %, p = 0.010). Survivors were overall more hemodynamically stable, as reflected by a lower need for mechanical circulatory support therapies, including VA-ECMO (16.1 % vs. 46 %, p < 0.001) and mAFP implantation (16.1 % vs. 54.0 %, p < 0.001).

**Table 2:** Laboratory biomarkers during ICU stay.

	**Survival(N = 118)**	**In-hospital death (N = 63)**	**All patients(N = 181)**	**P-value**
**Biomarkers assessed immediately after cardiac arrest**				
**pH**	7.25 (7.16–7.34)	7.12 (7.0–7.23)	7.22 (7.10–7.31)	<0.001
**Lactate (mg/dL)**	42.0 (20.5–65.5)	75.0 (53.0–97.0)	53.0 (26.5–78.3)	<0.001

**Blood count and coagulation markers**				
**Hemoglobin day 1 (g/dL)**	12.3 (±2.27)	11.3 (±2.32)	12.0 (±2.33)	0.004
**Hemoglobin day 2 (g/dL)**	11.0 (±2.14)	10.2 (±2.17)	10.7 (±2.17)	0.023
**Hemoglobin day 3 (g/dL)**	10.7 (±2.09)	9.54 (±1.91)	10.3 (±2.10)	<0.001
**Hemoglobin absolute change between days 1 and 3 (g/dL)**	−1.61 (±1.67)	−1.73 (±1.90)	−1.65 (±1.75)	0.667
**Hemoglobin relative change between days 1 and 3 (g/dL)**	−12.1 % (±13.9 %)	−13.5 % (±19.3 %)	−12.6 % (±15.9 %)	0.612
**Thrombocyte count day 1 (/nL)**	221 (±110)	186 (±89.6)	209 (±105)	0.026
**Thrombocyte count day 2 (/nL)**	178 (±86.6)	139 (±81.6)	164 (±86.7)	0.003
**Thrombocyte count day 3 (/nL)**	168 (±82.0)	115 (±72.4)	150 (±82.6)	<0.001
**Thrombocyte count absolute change between days 1 and 3 (per nL)**	−52.4 (±61.7)	−71.6 (±46.6)	−59.1 (±57.5)	0.020
**Thrombocyte count relative change between days 1 and 3 (per nL)**	–22.8 % (±20.3 %)	−37.9 % (±23.7 %)	−28.1 % (±22.7 %)	<0.001
**LDH day 1 (U/L)**	1120 (±1560)	1990 (±2020)	1430 (±1770)	0.004
**LDH day 2 (U/L)**	877 (±1050)	2290 (±1780)	1370 (±1500)	<0.001
**LDH day 3 (U/L)**	1560 (±2090)	2660 (±3330)	1940 (±2630)	0.020
**LDH absolute change between days 1 and 3 (U/L)**	437 (±2760)	668 (±3640)	518 (±3090)	0.660
**LDH relative change between days 1 and 3 (U/L)**	1130 % (±4390 %)	687 % (±3170 %)	975 % (±4010 %)	0.438

**Inflammatory markers**				
**Leukocyte count day 1 (/nL)**	13.8 (±5.41)	17.3 (±6.23)	15.0 (±5.93)	<0.001
**Leukocyte count day 2 (/nL)**	11.7 (±4.90)	15.5 (±5.56)	13.0 (±5.44)	<0.001
**Leukocyte count day 3 (/nL)**	10.7 (±4.45)	14.5 (±5.77)	12.0 (±5.26)	<0.001
**Leukocyte count absolute change between days 1 and 3 (/nL)**	−3.13 (±4.99)	−2.76 (±6.24)	−3.0 (±5.44)	0.683
**Leukocyte count relative change between days 1 and 3 (/nL)**	−16.4 % (±40.8 %)	−7.34 % (±48.1 %)	−13.2 % (±43.6 %)	0.207
**CRP day 1 (mg/L)**	74.0 (±91.4)	102 (±113)	83.6 (±100)	0.099
**CRP day 2 (mg/L)**	144 (±111)	183 (±107)	157 (±111)	0.023
**CRP day 3 (mg/L)**	148 (±125)	176 (±133)	158 (±128)	0.182
**CRP absolute change between days 1 and 3 (mg/L)**	74.2 (±150)	74.1 (±165)	74.2 (±155)	0.996
**CRP relative change between days 1 and 3 (mg/L)**	2390 % (±5130 %)	1260 % (±2670 %)	1990 % (±4460 %)	0.054

**Cardiac injury and perfusion**				
**CK day 1 (U/L)**	2410 (±2800)	7410 (±6960)	4150 (±5240)	<0.001
**CK day 2 (U/L)**	1990 (±2300)	6970 (±12400)	3720 (±7870)	0.002
**CK day 3 (U/L)**	1650 (±2360)	7780 (±16900)	3790 (±10500)	0.006
**CK absolute change between days 1 and 3 (U/L)**	−755 (±2550)	368 (±13600)	−364 (±8280)	0.520
**CK relative change between days 1 and 3 (U/L)**	48.4 % (±364 %)	20.0 % (±246 %)	38.5 % (±328 %)	0.535
**CK-MB day 1 (U/L)**	185 (±201)	580 (±484)	323 (±377)	<0.001
**Ck-MB day 2 (U/L)**	91.1 (±100)	294 (±296)	162 (±215)	<0.001
**CK-MB day 3 (U/L)**	50.3 (±55.0)	192 (±262)	99.6 (±174)	<0.001
**CK-MB absolute change between days 1 and 3 (U/L)**	−135 (±182)	−388 (±473)	–223 (±337)	<0.001
**CK-MB relative change between days 1 and 3 (U/L)**	−26.7 % (±211 %)	−53.9 % (±69.5 %)	−36.2 % (±175 %)	0.203
**Lactate day 1 (mg/dL)**	16.8 (±14.6)	35.0 (±26.7)	23.1 (±21.5)	<0.001
**Lactate day 2 (mg/dL)**	13.3 (±12.9)	30.9 (±30.3)	19.4 (±22.2)	<0.001
**Lactate day 3 (mg/dL)**	11.4 (±8.53)	28.3 (±33.7)	17.3 (±22.5)	<0.001
**Lactate absolute change between days 1 and 3 (mg/dL)**	−5.40 (±15.8)	−6.66 (±29.1)	−5.84 (±21.3)	0.750
**Lactate relative change between days 1 and 3 (mg/dL)**	−12.3 % (±73.3 %)	48.8 % (±525 %)	8.99 % (±315 %)	0.362

**Renal function indicators**				
**Creatinine day 1 (mg/dL)**	1.30 (±0.639)	1.86 (±1.12)	1.49 (±0.879)	<0.001
**Creatinine day 2 (mg/dL)**	1.32 (±0.845)	2.08 (±1.30)	1.59 (±1.09)	<0.001
**Creatinine day 3 (mg/dL)**	1.31 (±0.875)	2.17 (±1.44)	1.61 (±1.17)	<0.001
**Creatinine absolute change between days 1 and 3 (mg/dL)**	0.014 (±0.531)	0.308 (±0.858)	0.116 (±0.676)	0.015
**Creatinine relative change between days 1 and 3 (mg/dL)**	1.9 % (±38.5 %)	18.3 % (±55.2 %)	7.62 % (±45.6 %)	0.038
**GFR day 1 (ml/min)**	65.7 (±26.1)	46.1 (±22.6)	58.9 (±26.5)	<0.001
**GFR day 2 (ml/min)**	69.1 (±27.9)	46.4 (±28.0)	61.2 (±29.9)	<0.001
**GFR day 3 (ml/min)**	70.3 (±29.2)	43.2 (±27.5)	60.8 (±31.4)	<0.001
**GFR absolute change between days 1 and 3 (ml/min)**	4.58 (±22.6)	−2.92 (±22.4)	1.97 (±22.8)	0.035
**GFR relative change between days 1 and 3 (ml/min)**	12.9 % (±46.9 %)	−1.11 % (±56.5 %)	8.01 % (50.7 %)	0.096

**Metabolic parameters**				
**Glucose day 1 (mg/dL)**	142 (±38.5)	163 (±64.2)	150 (±49.9)	0.019
**Glucose day 2 (mg/dL)**	134 (±36.4)	158 (±52.6)	142 (±44.1)	0.002
**Glucose day 3 (mg/dL)**	136 (±38.7)	153 (±59.3)	142 (±47.5)	0.040
**Glucose absolute change between days 1 and 3 (mg/dL)**	−6.25 (±33.5)	−10.0 (±51.4)	−7.57 (±40.5)	0.598
**Glucose relative change between days 1 and 3 (mg/dL)**	−2.21 % (±24.2 %)	−1.77 % (±32.1 %)	−2.06 % (±27.1 %)	0.924
**Albumin day 1 (g/L)**	32.3 (±7.32)	26.4 (±6.91)	30.3 (±7.71)	<0.001
**Albumin day 2 (g/L)**	29.9 (±7.28)	25.2 (±7.03)	28.3 (±7.52)	<0.001
**Albumin day 3 (g/L)**	30.3 (±6.36)	24.6 (±7.94)	28.3 (±7.45)	<0.001
**Albumin absolute change between days 1 and 3 (g/L)**	−2.03 (±8.44)	−1.79 (±10.9)	−1.95 (±9.35)	0.883
**Albumin relative change between days 1 and 3 (g/L)**	−1.84 % (±29.5 %)	−0.09 % (±42.4 %)	−1.23 % (±34.4 %)	0.770
**Bilirubin day 1 (mg/dL)**	0.839 (±0.713)	0.755 (±0.597)	0.810 (±0.675)	0.402
**Bilirubin day 2 (mg/dL)**	0.984 (±1.06)	1.25 (±1.17)	1.08 (±1.11)	0.137
**Bilirubin day 3 (mg/dL)**	1.47 (±1.81)	1.50 (±1.89)	1.48 (±1.83)	0.934
**Bilirubin absolute change between days 1 and 3 (mg/dL)**	0.633 (±1.88)	0.741 (±1.91)	0.671 (±1.89)	0.717
**Bilirubin relative change between days 1 and 3 (mg/dL)**	251 % (±671 %)	207 % (±453 %)	235 % (±603 %)	0.606

**Neuronal injury markers**				
**NSE at hours 36 (µg/L)**	74.9 (±97.2)	138 (±141)	96.8 (±118)	0.002
**NSE at hours 72 (µg/L)**	101 (±153)	199 (±196)	135 (±175)	<0.001
**NSE absolute change between days 1 and 3 (µg/L)**	26.5 (±147)	61.3 (±181)	38.6 (±160)	0.193
**NSE relative change between days 1 and 3 (µg/L)**	233 % (±869 %)	234 % (±850 %)	233 % (±860 %)	0.993

**Electrolytes**				
**Sodium day 1 (mmol/L)**	140 (±2.99)	141 (±4.19)	141 (±3.45)	0.564
**Sodium day 2 (mmol/L)**	140 (±4.12)	142 (±5.37)	141 (±4.62)	0.080
**Sodium day 3 (mmol/L)**	142 (±3.80)	143 (±6.23)	142 (±4.82)	0.089
**Sodium absolute change between days 1 and 3 (mmol/L)**	1.50 (±3.64)	2.63 (±5.90)	1.90 (±4.57)	0.167
**Sodium relative change between days 1 and 3 (mmol/L)**	1.1 % (±2.61 %)	1.9 % (±4.21 %)	1.37 % (±3.27 %)	0.163
**Potassium day 1 (mmol/L)**	4.26 (±0.484)	4.66 (±0.638)	4.40 (±0.573)	<0.001
**Potassium day 2 (mmol/L)**	4.14 (±0.656)	4.51 (±0.673)	4.27 (±0.684)	<0.001
**Potassium day 3 (mmol/L)**	4.14 (±0.390)	4.51 (±0.882)	4.27 (±0.629)	0.003
**Potassium absolute change between days 1 and 3 (mmol/L)**	−0.118 (±0.632)	−0.151 (±0.980)	−0.129 (±0.768)	0.809
**Potassium relative change between days 1 and 3 (mmol/L**	−1.6 % (±14.1 %)	−2.1 % (±20.5 %)	−1.75 % (±16.5 %)	0.864
**Calcium day 1 (mmol/L)**	1.16 (±0.109)	1.17 (±0.079)	1.17 (±0.099)	0.600
**Calcium day 2 (mmol/L)**	1.16 (±0.073)	1.15 (±0.116)	1.16 (±0.090)	0.581
**Calcium day 3 (mmol/L)**	1.18 (±0.072)	1.16 (±0.111)	1.17 (±0.088)	0.185
**Calcium absolute change between days 1 and 3 (mmol/L)**	0.014 (±0.116)	−0.014 (±0.123)	0.005 (±0.119)	0.138
**Calcium relative change between days 1 and 3 (mmol/L)**	4.29 % (±38.7 %)	−0.85 % (±10.5 %)	2.50 % (±31.9 %)	0.178

CK; creatine kinase; CRP, C-reactive protein; GFR, glomerular filtration rate; LDH, lactate dehydrogenase; MB, muscle brain type; NSE, neuron specific enolase.

With respect to complications, survivors experienced significantly fewer complications than non-survivors. The most frequently observed adverse events were major bleeding, need for RRT, severe arrhythmias, sepsis and hemolysis. Survivors had a lower incidence of major bleeding (16.1 % vs. 31.7 %, p = 0.018) and were less likely to experience hemolysis (30.5 % vs. 61.9 %, p < 0.001), abdominal ischemia (2.5 % vs. 12.7 %, p = 0.016) and access-site ischemia (1.7 % vs. 14.3 %, p = 0.002). Survivors also had a significantly lower need for RRT (11.0 % vs. 50.8 %, p < 0.001) and were less likely to develop sepsis (8.5 % vs. 27.0 %, p = 0.002).

### Biomarker levels and in-hospital mortality

Patients who died had consistently lower hemoglobin and platelet levels on all three days following cardiac arrest, with a progressive worsening trend over time (Table 2). The optimal threshold for predicting mortality based on hemoglobin levels was identified at 11 g/dL (Table 3). An increase in hemoglobin level was associated with a substantial reduction in mortality risk, with an OR of 0.84 on day 1 and 0.73 on day 3. Furthermore, platelet counts decreased by 38 % in non-survivors compared to only 23 % in survivors over three days (p < 0.001), and a platelet count drop of 40 % within 72 h was identified as the critical cut-off value for predicting mortality risk. In addition, non-survivors demonstrated a progressive increase in hemolysis, as reflected by rising LDH levels from 1990 U/L to 2660 U/L over the three-day period, whereas survivors showed only a modest increase from 1120 U/L to 1560 U/L. However, despite these differences, LDH levels and their changes were not consistently associated with mortality in regression analyses.

**Table 3:** Effect of biomarker levels and their absolute or relative change within 72 h after cardiac arrest on in-hospital mortality (n = 181).

	**Optimal cut-off value**	**Odds ratio [95 % CI], p-value**
**Blood count and coagulation markers**		
**Hemoglobin day 1**	**11.7 g/dL**	**0.84 [0.71**–**1.04], p = 0.029**
**Hemoglobin day 3**	**10.6 g/dL**	**0.73 [0.60**–**0.88], p = 0.001**
**Hemoglobin absolute change between days 1 and 3**	−1.9 g/dL	0.89 [0.73–1.08], p = 0.230
**Hemoglobin relative change between days 1 and 3**	−15.3 %	0.18 [0.02–1.50], P = 0.110
**Thrombocyte count day 1**	118/nL	1.0 [0.99–1.0], p = 0.061
**Thrombocyte count day 3**	**127/nL**	**0.99 [0.98**–**0.99], p < 0.001**
**Thrombocyte count absolute change between days 1 and 3**	−38 /nL	0.99 [0.99–1.0], p = 0.051
**Thrombocyte count relative change between days 1 and 3**	**−39.8 %**	**0.05 [0.01**–**0.25], p = 0.001**
**LDH day 1**	**646 U/L**	**1.0 [1.0**–**1.0], p = 0.024**
**LDH day 3**	949 U/L	1.0 [1.0–1.0], p = 0.054
**LDH absolute change between days 1 and 3**	−51 U/L	1.0 [1.0–1.0], p = 0.665
**LDH relative change between days 1 and 3**	25.3 %	1.0 [0.99–1.01], p = 0.930

**Inflammatory markers**		
**Leukocyte count day 1**	**14.5 /nL**	**1.09 [1.03**–**1.16], p = 0.003**
**Leukocyte count day 3**	**11.2 /nL**	**1.15 [1.08**–**1.24], p < 0.001**
**Leukocyte count absolute change between days 1 and 3**	−5.18 /nL	1.02 [0.96–1.09], p = 0.517
**Leukocyte count relative change between days 1 and 3**	3.19 %	1.72 [0.80–3.66], p = 0.147
**CRP day 1**	3.5 mg/L	1.0 [1.0–1.01], p = 0.072
**CRP day 3**	209 mg/L	1.0 [1.0–1.0], p = 0.327
**CRP absolute change between days 1 and 3**	−53.2 mg/L	1.0 [1.0–1.0], p = 0.700
**CRP relative change between days 1 and 3**	**222 %**	**0.99 [0.98**–**1.0], p = 0.041**

**Cardiac injury and perfusion**		
**CK day 1**	**2257 U/L**	**1.0 [1.0**–**1.0], p < 0.001**
**CK day 3**	**866 U/L**	**1.0 [1.0**–**1.0], p = 0.006**
**CK absolute change between days 1 and 3**	−2204 U/L	1.0 [1.0–1.0], p = 0.943
**CK relative change between days 1 and 3**	−81.5 %	0.96 [0.83–1.07], p = 0.475
**CK-MB day 1**	**203 U/L**	**1.0 [1.0**–**1.01], p < 0.001**
**CK-MB day 3**	**51.3 U/L**	**1.01 [1.01**–**1.02], p < 0.001**
**CK-MB absolute change between days 1 and 3**	**−177 U/L**	**1.0 [1.0**–**1.0], p < 0.001**
**CK-MB relative change between days 1 and 3**	−70.9 %	0.87 [0.55–1.16], p = 0.467
**Lactate day 1**	**24 mg/dL**	**1.05 [1.03**–**1.07], p < 0.001**
**Lactate day 3**	**11 mg/dL**	**1.08 [1.04**–**1.13], p < 0.001**
**Lactate absolute change between days 1 and 3**	−10 mg/dL	1.0 [0.98–1.01], p = 0.734
**Lactate relative change between days 1 and 3**	−59.3 %	1.14 [0.98–1.87], p = 0.469

**Renal function indicators**		
**Creatinine day 1**	**1.42 mg/dL**	**2.14 [1.28**–**3.68], p = 0.005**
**Creatinine day 3**	**1.36 mg/dL**	**1.92 [1.37**–**2.79], p < 0.001**
**Creatinine absolute change between days 1 and 3**	**0.13 mg/dL**	**2.19 [1.32**–**3.84], p = 0.004**
**Creatinine relative change between days1 and 3**	**13.7 %**	**2.92 [1.40**–**6.59], p = 0.006**
**GFR day 1**	**63 ml/min**	**0.97 [0.96**–**0.99], p = 0.001**
**GFR day 3**	**65 ml/min**	**0.97 [0.96**–**0.98], p < 0.001**
**GFR absolute change between days 1 and 3**	**−4 ml/min**	**0.98 [0.96**–**0.99], p = 0.006**
**GFR relative change between days 1 and 3**	**−7.1 %**	**0.38 [0.16**–**0.81], p = 0.017**

**Metabolic parameters**		
**Glucose day 1**	**134 mg/dL**	**1.01 [1.0**–**1.02], p = 0.008**
**Glucose day 3**	**132 mg/dL**	**1.01 [1.0**–**1.02], p = 0.020**
**Glucose absolute change between days 1 and 3**	−33 mg/dL	1.0 [0.99–1.01], p = 0.463
**Glucose relative change between days 1 and 3**	−16.8 %	0.99 [0.26–3.59], p = 0.991
**Albumin day 1**	**26 g/L**	**0.89 [0.84**–**0.94], p < 0.001**
**Albumin day 3**	**25.4 g/L**	**0.89 [0.85**–**0.94], p < 0.001**
**Albumin absolute change between days 1 and 3**	0.9 g/L	1.0 [0.96–1.03], p = 0.826
**Albumin relative change between days 1 and 3**	−28.8 %	0.98 [0.38–2.56], p = 0.969
**Bilirubin day 1**	0.79 mg/dL	0.82 [0.47–1.39], p = 0.480
**Bilirubin day 3**	0.34 mg/dL	1.09 [0.89–1.32], p = 0.402
**Bilirubin absolute change between days 1 and 3**	0.16 mg/dL	1.10 [0.92–1.33], p = 0.296
**Bilirubin relative change between days 1 and 3**	66.7 %	1.0 [0.94–1.06], p = 0.977

**Neuronal injury markers**		
**NSE day 1**	**40.3 µg/L**	**1.0 [1.0**–**1.01], p = 0.014**
**NSE day 3**	**42 µg/L**	**1.0 [1.0**–**1.01], p = 0.001**
**NSE absolute change between days 1 and 3**	12.6 µg/L	1.0 [1.0–1.0], p = 0.061
**NSE relative change between days 1 and 3**	13.6 %	1.0 [0.96–1.04], p = 0.942

**Electrolytes**		
**Sodium day 1**	144 mmol/L	1.07 [0.97–1.19], p = 0.165
**Sodium day 3**	141 mmol/L	1.05 [0.98–1.14], p = 0.173
**Sodium absolute change between days 1 and 3**	0 mmol/L	1.02 [0.94–1.10], p = 0.690
**Sodium relative change between days 1 and 3**	0	9.57 [NA], p = 0.688
**Potassium day 1**	**4.3 mmol/L**	**2.94 [1.54**–**5.96], p = 0.002**
**Potassium day 3**	**4.3 mmol/L**	**3.17 [1.61**–**7.03], p = 0.002**
**Potassium absolute change between days 1 and 3**	−0.5 mmol/L	1.09 [0.70–1.71], p = 0.703
**Potassium relative change between days 1 and 3**	0	1.74 [0.22–14.52], p = 0.598
**Calcium day 1**	1.14 mmol/L	6.47 [NA], p = 0.396
**Calcium day 3**	1.12 mmol/L	0.03 [0.0–1.32], p = 0.075
**Calcium absolute change between days 1 and 3**	**−0.05 mmol/L**	**0.02 [0.0**–**0.64], p = 0.036**
**Calcium relative change between days 1 and 3**	−5.0 %	0.01 [0.0–0.77], p = 0.48

The multivariable regression analysis was adjusted for age, sex, “low-flow” time, initial arrest rhythm and location of cardiac arrest. The optimal cut-off values were calculated using the Youden-Index. CK, Creatinine-Kinase; CI, Confidence Interval; CK, creatine kinase; CK-MB, creatinine-kinase muscle brain type; CRP, C-reactive protein; GFR, glomerular filtration rate; LDH, lactate dehydrogenase; NSE, neuron-specific enolase.

Deceased patients also exhibited severe leukocytosis on day 1 (17 vs. 14 per nL, p < 0.001), which persisted but decreased slightly by day 3 (15 vs. 11 per nL, p < 0.001). Importantly, elevated leukocyte counts were strong predictors of mortality, with each unit increase in leukocyte count associated with a 9 % and 15 % increase in mortality risk on days 1 and 3, respectively.

Regarding organ injury, multiple domains were affected: First, cardiac injury was markedly greater in deceased patients, with CK levels of 7410 U/L compared to 2410 U/L in survivors on day 1 (p < 0.001). By day 3, CK levels further continued to increase in deceased patients (7780 U/L) but had decreased significantly in survivors (1650 U/L, p = 0.006). Similarly, CK-MB levels were approximately three to four times higher in deceased patients compared to survivors throughout the first three days (each p < 0.001). Both CK and CK-MB levels on day 1 and day 3 were independent predictors of mortality. Second, systemic perfusion impairment was reflected by significantly higher lactate levels in deceased patients, almost double those observed in survivors on all three days (each p < 0.001). An increase in lactate by one point was associated with a 5 % increase in mortality on day 1 and an 8 % increase on day 3. Optimal lactate cut-offs for mortality prediction were identified at 24 mg/dL on day 1 and 11 mg/dL on day 3. Third, renal dysfunction was significantly more pronounced in deceased patients, with a worsening trend over three days, whereas renal function remained relatively stable in survivors (creatinine increased by 18 % in non-survivors vs. 2 % in survivors, p = 0.038). Deceased patients also consistently had higher potassium levels (each p < 0.01). Regression analyses revealed that elevated creatinine levels were strongly associated with mortality (OR 2.14 on day 1 and OR 1.92 on day 3), with an optimal prediction threshold of 1.4 mg/dL. Likewise, elevated potassium levels were associated with 194 % higher mortality on day 1 and 217 % higher mortality on day 3. Fourth, non-survivors displayed metabolic derangements, with significantly higher glucose levels (up to 163 mmol/L), while survivors achieved better glycemic control by day 3 (136 mg/dL). Hyperglycemia was identified as an independent predictor of mortality, with optimal glucose thresholds for mortality prediction between 132 and 134 mg/dL. Hypoalbuminemia was also more severe in deceased patients, with albumin levels consistently around 25 g/L, compared to stable levels between 30 and 32 g/L in survivors (each p < 0.001). Albumin levels were significant mortality predictors, with a target threshold for prediction of 25–26 g/L. Fifth, neurological injury was more pronounced in deceased patients, who had nearly double the levels of NSE at both 36 and 72 h post-arrest (138 vs. 75 µg/L and 199 vs. 101 µg/L, respectively).

Subgroup analyses of patients who did not receive VA-ECMO therapy and those treated with VA-ECMO alone revealed robust findings. The majority of the previously identified independent predictors of in-hospital mortality remained significant in both subgroups (Supplemental Tables 1 and 2).

### Timing of biomarker levels assessment and in-hospital mortality

**Fig. 2:**
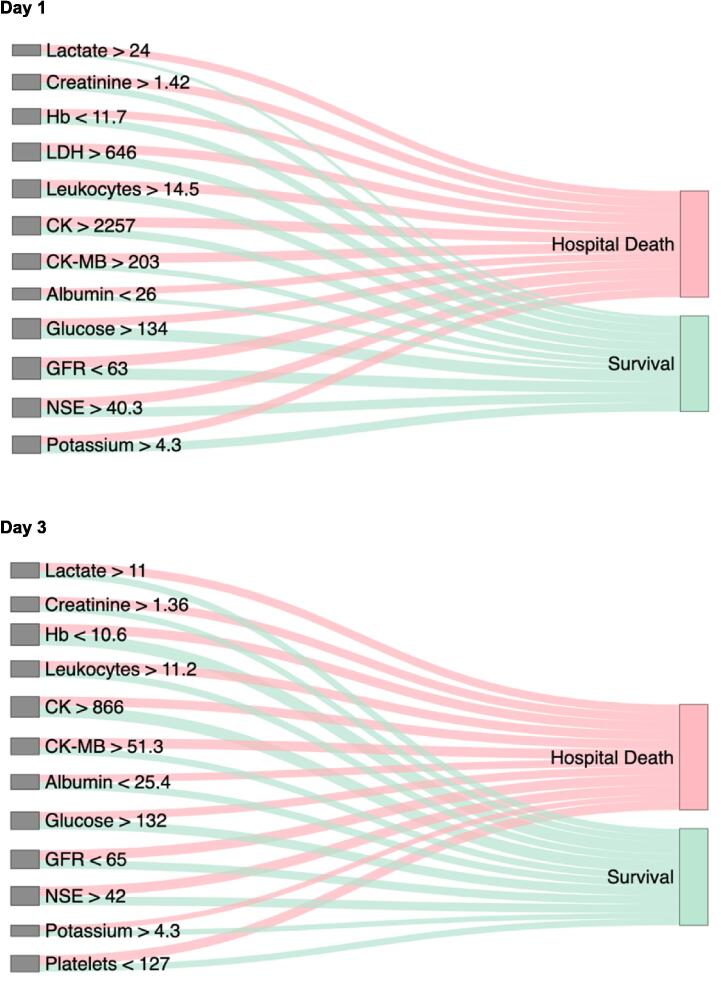
Sankey plots of biomarker levels from survivors and non-survivors on days one and three after cardiac arrest. The Sankey plots illustrate the distribution of biomarker levels in patients following infarct-related cardiac arrest, stratified by in-hospital survival status. Biomarkers on the left are dichotomized using threshold values derived from the *Youden index*, while the right side displays corresponding survival outcomes. Each flow line represents the number of patients with biomarker values above or below the respective cut-off, with line width proportional to the number of patients within each category. Units for each biomarker threshold are detailed in Table 3. CK, creatine kinase; GFR, glomerular filtration rate; Hb, hemoglobin; LDH, lactate-dehydrogenase; NSE, neuron-specific enolase. Units: albumin in g/L, CK in U/L, CK-MB in U/L, creatinine in mg/dL, GFR in ml/min, glucose in mg/dL, hemoglobin in g/dL, lactate in mg/dL, LDH in U/L, leukocytes per nL, NSE in µg/L, potassium in mmol/L, platelets per nL.

The stochastic neighbor embedded cluster plot analyses revealed that grouping all assessed biomarkers on day one following cardiac arrest did not effectively discriminate between survivors and non-survivors, due to a substantial overlap in biomarker profiles. However, by day three, a clearer separation emerged, forming two more distinct clusters based on the assessed biomarkers, indicating a divergence in physiological trajectories. Correspondingly, the radar plots demonstrated that on day one, the biomarker profiles of survivors and non-survivors were relatively close, with minimal separation. By day three, however, these profiles diverged more markedly, allowing for a clearer distinction between the two groups (Central Illustration). The Sankey plot analysis for day one illustrated that while early changes in biomarker profiles were already apparent, they remained relatively balanced and not distinctly separated between survivors and non-survivors. By day three, however, these differences became markedly more pronounced (Fig. 2). Individual trend analyses of key biomarkers showed that deceased patients exhibited a progressive dysregulation of markers such as LDH, creatinine, CK, albumin and glucose over the three-day period. In contrast, survivors demonstrated progressive stabilization and regulation of these same biomarkers (Supplemental Fig. 1).

## Discussion

This study analyzed dynamic biomarker trajectories within the first 72 h after AMI-induced cardiac arrest. While early biomarkers may guide therapeutic decisions, a clear prognostic separation between survivors and non-survivors did not emerge until day three, highlighting the need for serial, organ-specific monitoring over reliance on static admission-based variables. These findings support a shift toward time-dependent, multi-organ prognostic models for individualized post-resuscitation care.

### Dynamic trajectories and limitations of early prognostication

This study underscores the highly dynamic nature of patient trajectories in the first 72 h after cardiac arrest due to AMI. In cluster plot visualizations of the study cohort, survivors and non-survivors showed substantial overlap in biomarker profiles on day one, with only minimal separation, whereas by day three a much clearer divergence had emerged. This finding indicates that many patients who ultimately have different outcomes may appear physiologically similar in the immediate post-arrest period. Early outcome prognostication is therefore inherently challenging − a concept reflected in clinical observations that neurological prognosis is difficult to determine during the first 72 h post-arrest.^5^ Indeed, most widely used risk scores for post-cardiac arrest patients rely on clinical and laboratory parameters assessed at the pre-hospital or hospital admission level. While these scores (e.g., the “CAHP” or “MIRACLE_2_” score) provide an initial risk stratification based on crude variables such as age, time of arrest, initial arrest rhythm and initial blood pH, they cannot capture the subsequent evolution of a patient’s condition.^4^ The findings of this study caution against over-reliance on such early static assessments. Early biomarker levels and clinical scores may guide immediate ICU management but they should not serve as the sole basis for definitive prognostic decisions when patient trajectories are still in flux. In line with this, major recent guidelines, consensus statements and studies emphasize that early risk stratification is not to be used for decisions about withdrawal of life-sustaining therapy and “early” (<72 h) withdrawal of support is generally discouraged.^15^, ^16^, ^17^ Collectively, these findings emphasize that prognostication must be an ongoing process rather than a one-time judgment at admission.

### Multi-organ dysfunction in non-survivors versus restoration in survivors

A key finding of this study is that the post-resuscitation period represents a critical “second hit” that patients must survive following the initial cessation of cardiac activity, characterized by potential systemic inflammation, ischemia–reperfusion injury and multi-organ dysfunction. Non-survivors tended to develop progressive multi-organ failure in the days following resuscitation, whereas survivors showed gradual restoration of physiological homeostasis by day three. Non-survivors exhibited dysregulated levels of numerous organ-specific biomarkers, including markers of renal dysfunction (e.g., rising creatinine), cardiac injury (elevated CK and CK-MB), coagulopathy (progressive anemia, thrombocytopenia and hemolysis) and dysregulated metabolism (hyperglycemia and hypoalbuminemia). These trends point to an increasingly deranged systemic state in those who died, consistent with the development of multiple organ dysfunction syndrome. This pattern is consistent with the pathophysiology of post-cardiac arrest syndrome, in which global ischemia and reperfusion provoke systemic inflammation and microcirculatory dysfunction that can impair multiple organs.^18^ For instance, inadequate recovery of renal perfusion often manifested as worsening acute kidney injury; in this study, survivors frequently showed a decline in serum creatinine over 72 h, whereas non-survivors had persistently elevated or increasing creatinine. Notably, acute kidney injury in the early phase after cardiac arrest has been associated with worse outcomes in a substudy of the BOX trial.^19^ Similarly, resolution of lactic acidosis as well as normalization of hemostasis and coagulation parameters were observed predominantly in survivors, suggesting an abatement of post-resuscitation shock and inflammatory coagulopathy in those who recovered.^20^ In contrast, the persistence of hemostasis and coagulopathy in non-survivors is in agreement with reports that post-arrest disseminated intravascular coagulation and related derangements are linked to poorer outcomes.^21^, ^22^ Taken together, these observations illustrate that survivors, despite critical illness initially, were able to re-equilibrate their internal milieu within the first 72 h, whereas non-survivors experienced a cascade of secondary organ failures. This dichotomy was only fully appreciable with serial measurements over time, reinforcing the importance of early systemic monitoring of organ function after cardiac arrest.

### Integrating serial biomarkers into prognostication

In summary, this study supports an early and comprehensive monitoring strategy in post-cardiac arrest care, one that includes serial biomarker assessment across multiple organ systems. In this study cohort, multiple laboratory values on both day one and day three were identified that were individually predictive of in-hospital mortality, underscoring that no single biomarker or clinical feature is sufficient for accurate prognostication alone. This multifactorial reality highlights the need to integrate diverse data points. For example, combining neurological indicators with organ-specific biomarkers may substantially improve prognostic accuracy. Neurologic exams and imaging remain paramount for assessing hypoxic brain injury, and markers such as NSE are well-established aids in neuro-prognostication. However, these findings suggest that parallel tracking of extracerebral organ function is also critical. A patient’s neurological recovery can be modulated or even thwarted by failure of other organs, e.g. refractory shock due to cardiac dysfunction or renal failure causing metabolic derangements. Incorporating organ injury biomarkers (for heart, kidneys, liver, etc.) into prognostic models alongside traditional neurological endpoints should be considered. This approach is consistent with current expert recommendations for multimodal prognostication, which stress that no single test should be used in isolation and that a combination of clinical examination, physiology, imaging and biomarkers offers the most robust assessment.^16^ Approaches, such as machine learning, may facilitate the integration of multidimensional clinical and biomarker data in a user-friendly and reliable manner, as exemplified by the cluster plots in this study. These techniques offer the potential to enhance prognostic accuracy by capturing complex, non-linear relationships that may not be evident through traditional static approaches.

### Limitations

This single-center study may limit generalizability due to institutional differences in post-arrest care. Due to the exclusion of patients who died within the first 72 h, these findings primarily reflect prognostic uncertainty in patients with relatively favorable early clinical courses and may underestimate early severity in rapidly deteriorating patients (survivorship bias). These findings may not capture the full severity spectrum of early post-arrest organ failure and should be interpreted within this context. Generalizability to the broader arrest population, including non-AMI etiologies and patients with fulminant early decline, is limited. The observational design is subject to selection bias and unmeasured confounding and causal inference cannot be drawn. Finally, external validation in larger, multicenter cohorts is required to confirm the prognostic utility of the identified biomarker trajectories and their integration into dynamic risk models.

## Conclusion

In patients who survive the initial critical phase after cardiac arrest, early prognostication within the first 72 h post-cardiac arrest is limited by evolving patient trajectories. While biomarkers at admission may offer valuable guidance, they should not be used to make definitive decisions, as the early post-resuscitation period represents a critical “second hit” that significantly influences patient survival. Modern machine learning approaches offer a powerful and easy way to develop and deploy dynamic risk models by integrating complex, serial, organ-specific data, thereby capturing the evolving physiological landscape of patients following cardiac arrest. Survivors typically re-establish homeostasis, while non-survivors progress to multi-organ failure, emphasizing the importance of modern, multimodal and individualized monitoring strategies. Future studies should expand on these findings in more heterogeneous cardiac arrest populations, including those with non-coronary etiologies and earlier mortality, and should incorporate modern cardiac markers, such as high-sensitivity troponin, for enhanced sensitivity and early stratification.

## CRediT authorship contribution statement

**Julian Mohsennia:** Writing – review & editing, Writing – original draft, Visualization, Validation, Software, Methodology, Investigation, Formal analysis, Data curation. **Sophia Neschen:** Writing – review & editing, Writing – original draft, Validation, Software, Methodology, Investigation, Formal analysis, Data curation. **Joshua Boettel:** Writing – review & editing, Validation, Resources, Methodology, Investigation, Formal analysis, Data curation. **Steffen Desch:** Writing – review & editing, Methodology, Investigation, Data curation. **Youssef Abdelwahed:** Writing – review & editing, Resources, Methodology, Investigation, Data curation. **Tobias Petzold:** Writing – review & editing, Methodology, Investigation, Data curation. **Andi Rroku:** Writing – review & editing, Methodology, Investigation, Data curation. **Eva-Maria Dorsch:** Writing – review & editing, Resources, Methodology, Investigation, Data curation. **Georg Girke:** Writing – review & editing, Methodology, Investigation, Data curation. **Benjamin O’Brien:** Writing – review & editing, Resources, Methodology, Investigation, Data curation. **Ulf Landmesser:** Writing – review & editing, Writing – original draft, Resources, Methodology, Investigation, Data curation. **Carsten Skurk:** Writing – review & editing, Resources, Methodology, Investigation, Data curation. **Tharusan Thevathasan:** Writing – review & editing, Writing – original draft, Visualization, Validation, Supervision, Resources, Project administration, Methodology, Investigation, Data curation, Conceptualization.

## Funding

This research did not receive any specific grant from funding agencies in the public, commercial or not-for-profit sectors.

## Declaration of competing interest

None.
