# Supplementary material for: Dynamic biomarker trajectories in the first 72 h after infarct-related cardiac arrest: a novel approach to early risk stratification
Source: Resusc Plus. 2025 Oct 13;26:101126. doi: 10.1016/j.resplu.2025.101126 (PMC12615751; doi:10.1016/j.resplu.2025.101126)
Supplement: Supplementary Data 1 [file mmc1.docx]

**Dynamic Biomarker Trajectories Within 72 Hours After Infarct-Related Cardiac Arrest: A Time-Sensitive Approach to Early Risk Stratification**

**-**

**Supplemental Material**

***Supplemental Table 1.*** Effect of biomarker levels and their absolute or relative change within 72 hours after cardiac arrest on in-hospital mortality in patients not receiving VA-ECMO therapy (n=133)

|  | **Optimal cut-off value** | **Odds ratio [95% CI], p-value** |
| --- | --- | --- |
| **Blood count and coagulation markers** | | |
| **Hemoglobin day 1** | 13.2 g/dL | 0.89 [0.71 – 1.11], p = 0.317 |
| **Hemoglobin day 3** | **11.9 g/dL** | **0.72 [0.56 – 0.92], p = 0.011** |
| **Hemoglobin absolute change between days 1 and 3** | -1.9 g/dL | 0.76 [0.58 – 1.00], p = 0.054 |
| **Hemoglobin relative change between days 1 and 3** | **-15.3 %** | **0.01 [0.00 – 0.39], p = 0.018** |
| **Thrombocyte count day 1** | 246 /nL | 1.00 [0.99 – 1.00], p = 0.453 |
| **Thrombocyte count day 3** | **123 /nL** | **0.99 [0.98 – 1.00], p = 0.018** |
| **Thrombocyte count absolute change between days 1 and 3** | -38 /nL | 0.99 [0.99 – 1.00], p = 0.068 |
| **Thrombocyte count relative change between days 1 and 3** | **-25.0 %** | **0.01 [0.00 – 0.14], p = 0.001** |
| **LDH day 1** | 663 U/L | 1.00 [1.00 – 1.00], p = 0.122 |
| **LDH day 3** | 6918 U/L | 1.00 [1.00 – 1.00], p = 0.156 |
| **LDH absolute change between days  1 and 3** | -51 U/L | 1.00 [1.00 – 1.00], p = 0.691 |
| **LDH relative change between days  1 and 3** | -9.03 % | 0.99 [0.94 – 1.01], p = 0.455 |
| **Inflammatory markers** | | |
| **Leukocyte count day 1** | **14.5 /nL** | **1.10 [1.02 – 1.19], p = 0.015** |
| **Leukocyte count day 3** | **10.1 /nL** | **1.16 [1.05 – 1.29], p = 0.004** |
| **Leukocyte count absolute change between days 1 and 3** | -4.79 /nL | 1.00 [0.90 – 1.10], p = 0.944 |
| **Leukocyte count relative change between days 1 and 3** | -29.5 % | 1.45 [0.35 – 4.46], p = 0.552 |
| **CRP day 1** | 41.9 mg/L | 1.00 [1.00 – 1.01], p = 0.202 |
| **CRP day 3** | 227 mg/L | 1.00 [1.00 – 1.00], p = 0.562 |
| **CRP absolute change between days  1 and 3** | -53.2 mg/L | 1.00 [1.00 – 1.00], p = 0.733 |
| **CRP relative change between days 1 and 3** | 222 % | 0.99 [0.98 – 1.00], p = 0.110 |
| **Cardiac injury and perfusion** | | |
| **CK day 1** | **1640 U/L** | **1.00 [1.00 – 1.00], p < 0.001** |
| **CK day 3** | 1169 U/L | 1.00 [1.00 – 1.00], p = 0.213 |
| **CK absolute change between days 1 and 3** | -826 U/L | 1.00 [1.00 – 1.00], p = 0.518 |
| **CK relative change between days 1 and 3** | -81.5 % | 0.94 [0.79 – 1.07], p = 0.389 |
| **CK-MB day 1** | **203 U/L** | **1.00 [1.00 – 1.01], p < 0.001** |
| **CK-MB day 3** | **37 U/L** | **1.01 [1.00 – 1.02], p = 0.008** |
| **CK-MB absolute change between days  1 and 3** | **-162 U/L** | **1.00 [0.99 – 1.00], p = 0.007** |
| **CK-MB relative change between days  1 and 3** | -84.2 % | 0.83 [0.45 – 1.18], p = 0.426 |
| **Lactate day 1** | **24 mg/dL** | **1.04 [1.01 – 1.07], p = 0.008** |
| **Lactate day 3** | **11 mg/dL** | **1.05 [1.02 – 1.11], p = 0.016** |
| **Lactate absolute change between days  1 and 3** | 2 mg/dL | 1.01 [0.98 – 1.03], p = 0.494 |
| **Lactate relative change between days  1 and 3** | 18.2 % | 1.20 [0.97 – 2.25], p = 0.541 |
| **Renal function indicators** | | |
| **Creatinine day 1** | 1.47 mg/dL | 1.68 [0.94 – 3.28], p = 0.111 |
| **Creatinine day 3** | **1.36 mg/dL** | **1.70 [1.10 – 2.79], p = 0.026** |
| **Creatinine absolute change between days  1 and 3** | 0.24 mg/dL | 2.05 [0.99 – 4.50], p = 0.059 |
| **Creatinine relative change between days 1 and 3** | 10.7 % | 2.50 [0.85 – 7.31], p = 0.089 |
| **GFR day 1** | 45 ml/min | 0.98 [0.96 – 1.00], p = 0.075 |
| **GFR day 3** | **65 ml/min** | **0.98 [0.96 – 0.99], p = 0.004** |
| **GFR absolute change between days  1 and 3** | 2 ml/min | 0.98 [0.96 – 1.00], p = 0.056 |
| **GFR relative change between days 1 and 3** | 5.94 % | 0.34 [0.09 – 10.6], p = 0.083 |
| **Metabolic parameters** | | |
| **Glucose day 1** | **181 mg/dL** | **1.02 [1.01 – 1.03], p = 0.002** |
| **Glucose day 3** | 132 mg/dL | 1.01 [1.00 – 1.02], p = 0.092 |
| **Glucose absolute change between days  1 and 3** | **-33 mg/dL** | **0.98 [0.97 – 1.00], p = 0.013** |
| **Glucose relative change between days  1 and 3** | -6.93 % | 0.12 [0.01 – 0.91], p = 0.054 |
| **Albumin day 1** | **32.5 g/L** | **0.91 [0.86 – 0.97], p = 0.005** |
| **Albumin day 3** | **29.0 g/L** | **0.87 [0.81 – 0.93], p < 0.001** |
| **Albumin absolute change between days  1 and 3** | -11.7 g/L | 0.98 [0.93 – 1.02], p = 0.310 |
| **Albumin relative change between days  1 and 3** | -34.3 % | 0.49 [0.11 – 1.87], p = 0.310 |
| **Bilirubin day 1** | 0.66 mg/dL | 0.81 [0.41 – 1.53], p = 0.533 |
| **Bilirubin day 3** | 0.98 mg/dL | 0.70 [0.44 – 0.98], p = 0.069 |
| **Bilirubin absolute change between days  1 and 3** | 0.76 mg/dL | 0.79 [0.57 – 1.05], p = 0.128 |
| **Bilirubin relative change between days  1 and 3** | -42.2 % | 0.92 [0.79 – 1.01], p = 0.172 |
| **Neuronal injury markers** | | |
| **NSE day 1** | 40.3 µg/L | 1.00 [1.00 – 1.01], p = 0.106 |
| **NSE day 3** | **17.6 µg/L** | **1.00 [1.00 – 1.00], p = 0.017** |
| **NSE absolute change between days  1 and 3** | 12.6 µg/L | 1.00 [1.00 – 1.01], p = 0.092 |
| **NSE relative change between days 1 and 3** | -26.9 % | 0.98 [0.91 – 1.03], p = 0.452 |
| **Electrolytes** | | |
| **Sodium day 1** | 140 mmol/L | 0.99 [0.86 – 1.14], p = 0.864 |
| **Sodium day 3** | 150 mmol/L | 1.02 [0.91 – 1.14], p = 0.776 |
| **Sodium absolute change between days  1 and 3** | 1 mmol/L | 1.02 [0.92 – 1.15], p = 0.681 |
| **Sodium relative change between days  1 and 3** | -0.70 % | 27.07 [NA], p = 0.676 |
| **Potassium day 1** | 4.3 mmol/L | 2.08 [0.93 – 4.79], p = 0.076 |
| **Potassium day 3** | 4.5 mmol/L | 2.25 [1.00 – 5.65], p = 0.060 |
| **Potassium absolute change between days 1 and 3** | -0.5 mmol/L | 1.04 [0.56 – 1.94], p = 0.899 |
| **Potassium relative change between days  1 and 3** | -12.5 % | 1.52 [0.09 – 26.44], p = 0.773 |
| **Calcium day 1** | 1.15 mmol/L | 1.42 [NA], p = 0.883 |
| **Calcium day 3** | 1.17 mmol/L | 0.03 [0.00 – 4.77], p = 0.175 |
| **Calcium absolute change between days  1 and 3** | 0.07 mmol/L | 0.09 [0.00 – 4.44], p = 0.249 |
| **Calcium relative change between days  1 and 3** | 6.0 % | 0.08 [0.00 – 2.08], p = 0.330 |
| The multivariable regression analysis was adjusted for age, sex, “low-flow” time, initial arrest rhythm and location of cardiac arrest. The optimal cut-off values were calculated using the Youden-Index. CK, Creatinine-Kinase; CI, Confidence Interval; CK, creatine kinase; CK-MB, creatinine-kinase muscle brain type; CRP, C-reactive protein; GFR, glomerular filtration rate; LDH, lactate dehydrogenase; NSE, neuron-specific enolase; VA-ECMO, veno-arterial extracorporeal membrane oxygenation. | | |

***Supplemental Table 2.*** Effect of biomarker levels and their absolute or relative change within 72 hours after cardiac arrest on in-hospital mortality in patients receiving VA-ECMO therapy (n=48)

|  | |  | **Optimal**  **cut-off value** | **Odds ratio**  **[95% CI], p-value** |  |
| --- | --- | --- | --- | --- | --- |
|  | **Blood count and coagulation markers** | | | | |
| **Hemoglobin day 1** | |  | 10.2 g/dL | 1.14 [0.78 – 1.71], p = 0.513 |  |
| **Hemoglobin day 3** | |  | 7.8 g/dL | 1.40 [0.85 – 2.57], p = 0.222 |  |
| **Hemoglobin absolute change between days 1 and 3** | |  | -3.2 g/dL | 1.06 [0.75 – 1.52], p = 0.747 |  |
| **Hemoglobin relative change between days 1 and 3** | |  | **-31.1 %** | 2.49 [NA], p = 0.598 |  |
| **Thrombocyte count day 1** | |  | 113 /nL | 1.00 [0.99 – 1.01], p = 0.656 |  |
| **Thrombocyte count day 3** | |  | 69 /nL | 0.99 [0.98 – 1.00], p = 0.240 |  |
| **Thrombocyte count absolute change between days 1 and 3** | |  | -46 /nL | 0.99 [0.98 – 1.01], p = 0.449 |  |
| **Thrombocyte count relative change between days 1 and 3** | |  | **-42.5 %** | 0.46 [0.03 – 7.21], p = 0.572 |  |
| **LDH day 1** | |  | **2431 U/L** | **1.00 [1.00 – 1.00], p = 0.029** |  |
| **LDH day 3** | |  | 949 U/L | 1.00 [1.00 – 1.00], p = 0.123 |  |
| **LDH absolute change between days  1 and 3** | |  | -1636 U/L | 1.00 [1.00 – 1.00], p = 0.655 |  |
| **LDH relative change between days  1 and 3** | |  | -34.6 % | 1.02 [0.99 – 1.14], p = 0.562 |  |
|  | **Inflammatory markers** | | | | |
| **Leukocyte count day 1** | |  | 18.5 /nL | 1.07 [0.96 – 1.21], p = 0.245 |  |
| **Leukocyte count day 3** | |  | **12.0 /nL** | 1.04 [0.90 – 1.20], p = 0.610 |  |
| **Leukocyte count absolute change between days 1 and 3** | |  | -5.53 /nL | 0.96 [0.85 – 1.07], p = 0.438 |  |
| **Leukocyte count relative change between days 1 and 3** | |  | 6.64 % | 0.91 [0.24 – 3.41], p = 0.882 |  |
| **CRP day 1** | |  | 119 mg/L | 1.00 [1.00 – 1.01], p = 0.223 |  |
| **CRP day 3** | |  | 209 mg/L | 1.00 [1.00 – 1.01], p = 0.210 |  |
| **CRP absolute change between days  1 and 3** | |  | 117 mg/L | 1.00 [1.00 – 1.01], p = 0.841 |  |
| **CRP relative change between days 1 and 3** | |  | 1293 % | 0.98 [0.93 – 1.02], p = 0.376 |  |
|  | **Cardiac injury and perfusion** | | | | |
| **CK day 1** | |  | **10445 U/L** | **1.00 [1.00 – 1.00], p = 0.016** |  |
| **CK day 3** | |  | 5382 U/L | 1.00 [1.00 – 1.00], p = 0.130 |  |
| **CK absolute change between days 1 and 3** | |  | -2519 U/L | 1.00 [1.00 – 1.00], p = 0.191 |  |
| **CK relative change between days 1 and 3** | |  | -61.1 % | 1.55 [0.87 – 3.43], p = 0.187 |  |
| **CK-MB day 1** | |  | **777 U/L** | **1.00 [1.00 – 1.01], p = 0.006** |  |
| **CK-MB day 3** | |  | **143 U/L** | **1.02 [1.00 – 1.03], p = 0.035** |  |
| **CK-MB absolute change between days  1 and 3** | |  | -655 U/L | 1.00 [1.00 – 1.00], p = 0.072 |  |
| **CK-MB relative change between days  1 and 3** | |  | -78.3 % | 3.75 [NA], p = 0.209 |  |
| **Lactate day 1** | |  | **32 mg/dL** | **1.05 [1.01 – 1.10], p = 0.025** |  |
| **Lactate day 3** | |  | **17 mg/dL** | **1.31 [1.12 – 1.65], p = 0.006** |  |
| **Lactate absolute change between days  1 and 3** | |  | -20 mg/dL | 1.00 [0.97 – 1.02], p = 0.850 |  |
| **Lactate relative change between days  1 and 3** | |  | 50.0 % | 2.03 [0.50 – 10.87], p = 0.356 |  |
|  | **Renal function indicators** | | | | |
| **Creatinine day 1** | |  | 1.97 mg/dL | 2.84 [1.02 – 9.45], p = 0.061 |  |
| **Creatinine day 3** | |  | 1.12 mg/dL | 1.72 [0.97 - 3.42], p = 0.086 |  |
| **Creatinine absolute change between days  1 and 3** | |  | 0.13 mg/dL | 1.54 [0.68 – 3.90], p = 0.316 |  |
| **Creatinine relative change between days 1 and 3** | |  | 15.4 % | 2.60 [0.70 – 16.08], p = 0.216 |  |
| **GFR day 1** | |  | **45 ml/min** | **0.96 [0.92 – 0.99], p = 0.022** |  |
| **GFR day 3** | |  | **56 ml/min** | **0.97 [0.94 – 0.99], p = 0.014** |  |
| **GFR absolute change between days  1 and 3** | |  | 0 ml/min | 0.98 [0.94 – 1.01], p = 0.209 |  |
| **GFR relative change between days 1 and 3** | |  | -5.05 % | 0.67 [0.18 – 2.49], p = 0.514 |  |
|  | **Metabolic parameters** | | | | |
| **Glucose day 1** | |  | 188 mg/dL | 1.00 [0.99 – 1.02], p = 0.810 |  |
| **Glucose day 3** | |  | 173 mg/dL | 1.01 [0.99 – 1.03], p = 0.292 |  |
| **Glucose absolute change between days  1 and 3** | |  | **8 mg/dL** | 1.01 [0.99 – 1.03], p = 0.319 |  |
| **Glucose relative change between days  1 and 3** | |  | 2.64 % | 4.41 [NA], p = 0.249 |  |
| **Albumin day 1** | |  | **25.4 g/L** | **0.82 [0.69 – 0.94], p = 0.009** |  |
| **Albumin day 3** | |  | **25.4 g/L** | 0.95 [0.87 – 1.04], p = 0.282 |  |
| **Albumin absolute change between days  1 and 3** | |  | 10.4 g/L | 1.03 [0.96 – 1.11], p = 0.432 |  |
| **Albumin relative change between days  1 and 3** | |  | 31.1 % | 2.91 [0.51 – 20.90], p = 0.249 |  |
| **Bilirubin day 1** | |  | 0.79 mg/dL | 0.87 [0.22 – 3.49], p = 0.842 |  |
| **Bilirubin day 3** | |  | 2.32 mg/dL | **2.16 [1.26 – 4.59], p = 0.017** |  |
| **Bilirubin absolute change between days  1 and 3** | |  | 1.31 mg/dL | **1.94 [1.21 – 3.58], p = 0.014** |  |
| **Bilirubin relative change between days  1 and 3** | |  | 201 % | 1.26 [1.03 – 1.68], p = 0.055 |  |
|  | **Neuronal injury markers** | | | | |
| **NSE day 1** | |  | 109 µg/L | 1.01 [1.00 – 1.02], p = 0.084 |  |
| **NSE day 3** | |  | **43.6 µg/L** | **1.02 [1.00 – 1.04], p = 0.042** |  |
| **NSE absolute change between days  1 and 3** | |  | 23.6 µg/L | 1.00 [1.00 – 1.01], p = 0.131 |  |
| **NSE relative change between days 1 and 3** | |  | 14.3 % | 1.41 [1.01 – 2.79], p = 0.194 |  |
|  | **Electrolytes** | | | | |
| **Sodium day 1** | |  | 146 mmol/L | 1.06 [0.89 – 1.28], p = 0.537 |  |
| **Sodium day 3** | |  | 139 mmol/L | 0.99 [0.87 – 1.12], p = 0.824 |  |
| **Sodium absolute change between days  1 and 3** | |  | 4 mmol/L | 0.95 [0.83 – 1.09], p = 0.464 |  |
| **Sodium relative change between days  1 and 3** | |  | 2.80 % | 0.00 [NA], p = 0.492 |  |
| **Potassium day 1** | |  | 4.6 mmol/L | 3.98 [1.01 – 21.34], p = 0.073 |  |
| **Potassium day 3** | |  | 4.3 mmol/L | 3.98 [1.02 – 35.37], p = 0.129 |  |
| **Potassium absolute change between days 1 and 3** | |  | -0.8 mmol/L | 1.12 [0.55 – 2.67], p = 0.767 |  |
| **Potassium relative change between days  1 and 3** | |  | 11.7 % | 1.91 [NA], p = 0.733 |  |
| **Calcium day 1** | |  | 1.20 mmol/L | 7.67 [NA], p = 0.647 |  |
| **Calcium day 3** | |  | 1.09 mmol/L | 0.01 [0.00 – 17.78], p = 0.222 |  |
| **Calcium absolute change between days  1 and 3** | |  | 0.02 mmol/L | 0.00 [0.00 – 4.55], p = 0.144 |  |
| **Calcium relative change between days  1 and 3** | |  | 17.4 % | 0.00 [0.00 – 5.22], p = 0.142 |  |
| The multivariable regression analysis was adjusted for age, sex, “low-flow” time, initial arrest rhythm and location of cardiac arrest. The optimal cut-off values were calculated using the Youden-Index. CK, Creatinine-Kinase; CI, Confidence Interval; CK, creatine kinase; CK-MB, creatinine-kinase muscle brain type; CRP, C-reactive protein; GFR, glomerular filtration rate; LDH, lactate dehydrogenase; NSE, neuron-specific enolase; VA-ECMO, veno-arterial extracorporeal membrane oxygenation. | | | | |  |

***Supplemental Figure 1.*** Trajectories of biomarker levels within 72 hours after cardiac arrest between survivors and non-survivors


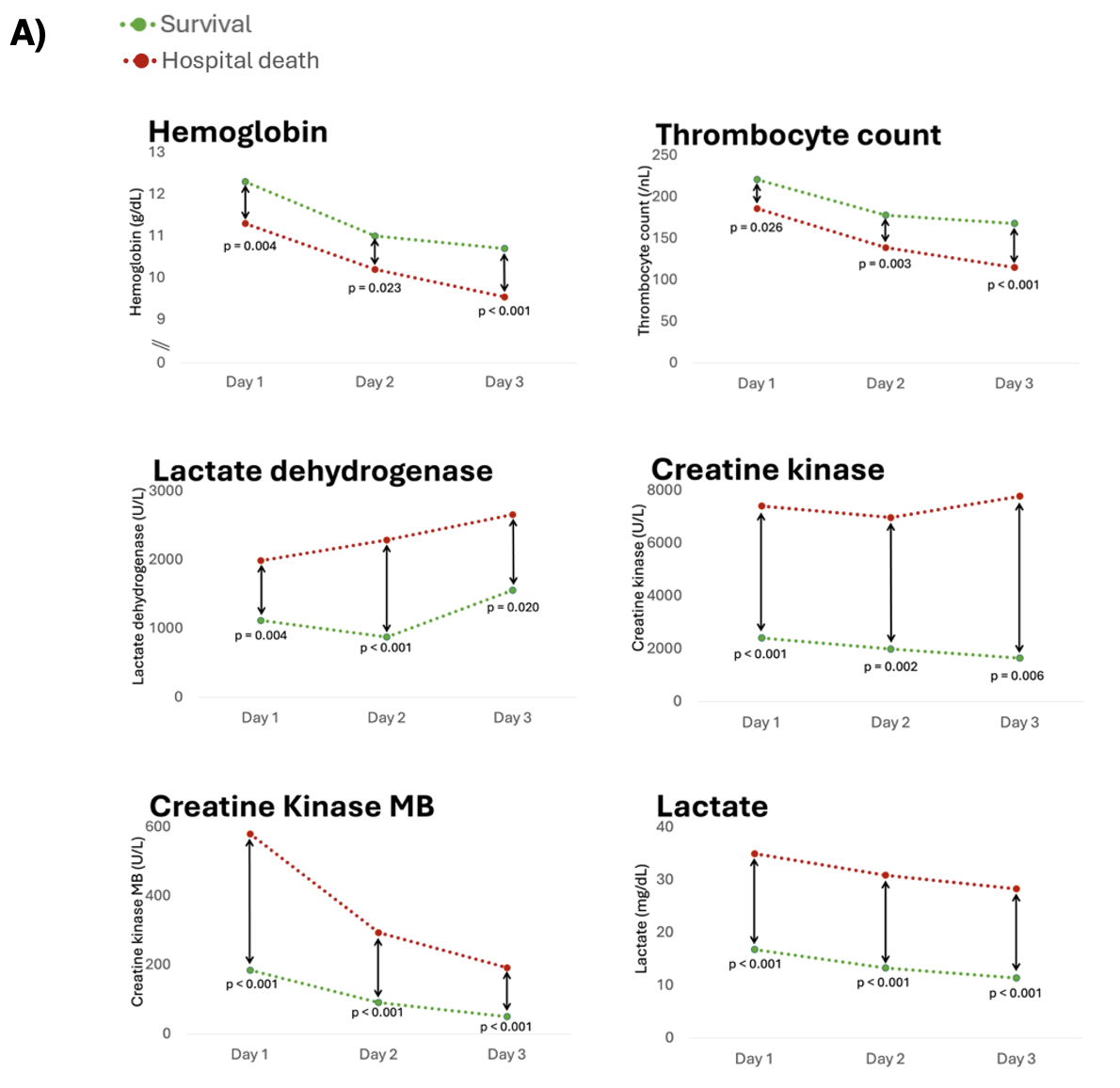


**
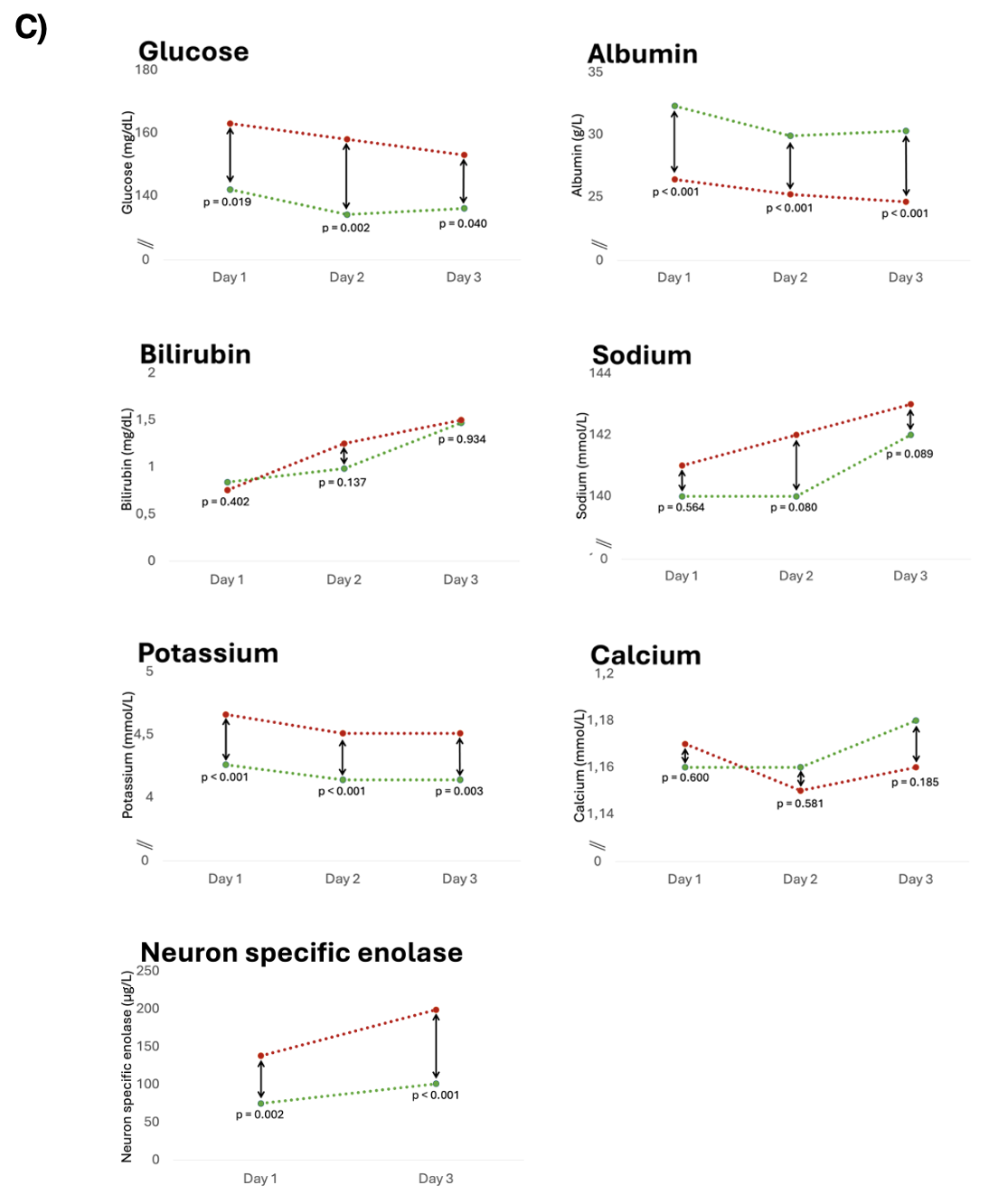
**

**
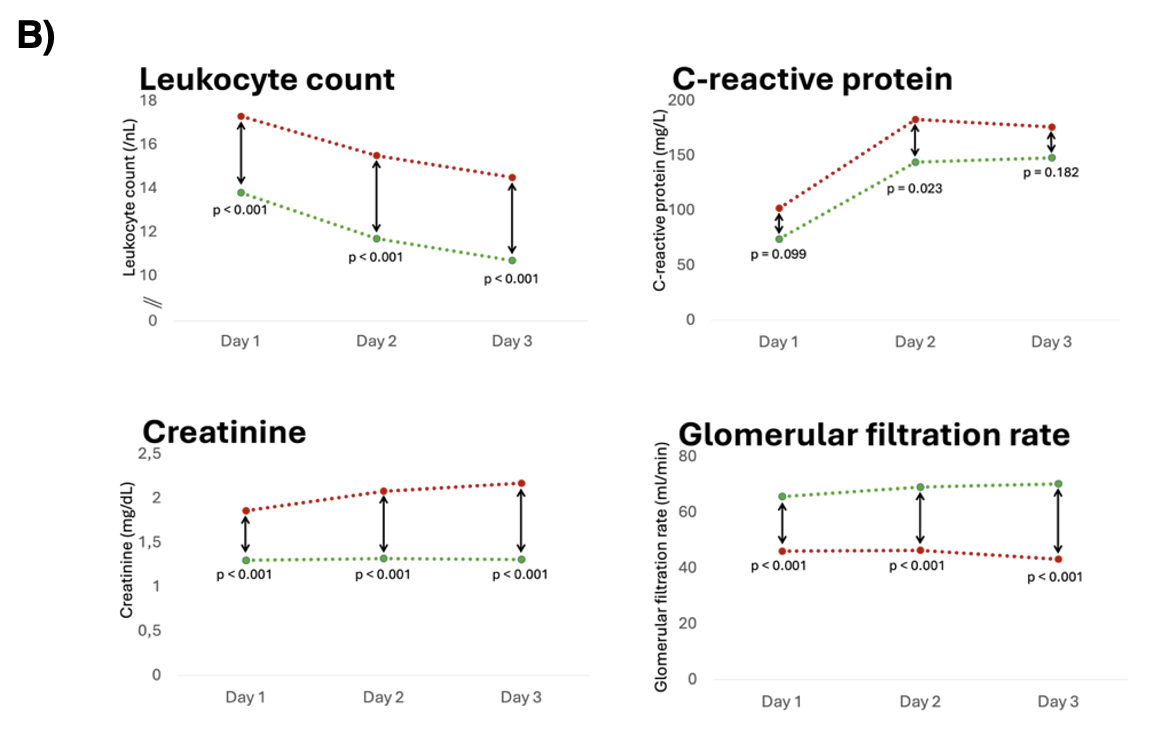
**

**Figure legend:** Temporal trajectories of biomarker levels over the first three days following cardiac arrest are illustrated, stratified by survivors (green) and non-survivors (red). At each time point, comparisons between the two groups were made using two-sample t-tests to assess statistically significant differences in biomarker levels.
